# Intuition, reflection, and prosociality: Evidence from a field experiment

**DOI:** 10.1371/journal.pone.0262476

**Published:** 2022-02-25

**Authors:** Sascha Grehl, Andreas Tutić

**Affiliations:** Institut für Soziologie, Leipzig University, Leipzig, Saxony, Germany; Universidad Loyola Andalucia Cordoba, SPAIN

## Abstract

Are humans instinctively good or is it only our capacity for reflection that enables us to restrain our selfish traits and behave prosocially? Against the background of dual-process theory, the question of whether people tend to behave prosocially on intuitive grounds has been debated controversially for several years. Central to this debate is the so-called social heuristic hypothesis (SHH), which states that subjects orient their behavior more closely to their deeply ingrained norms and attitudes when the behavior comes about in an intuitive rather than reflective manner. In this paper, we apply the SHH to a novel setting and investigate whether its implications hold true in a non-reactive field experiment, in which subjects are unaware that they are part of a study. We test whether subjects report a misdirected email or try to use the opportunity to reap a monetary benefit. Since all subjects participated six months prior to the field experiment in a lab experiment, we have solid measures of the subjects’ general tendency to behave intuitively and their prosocial attitudes. In addition, participants were asked in a follow-up survey to self-report their intuitiveness at the time of the decision. While we observe a significant and positive effect on prosocial behavior for self-reported intuitiveness (but not for general intuitiveness) in the bivariate analyses, this effect becomes insignificant when controlling for interaction effects with attitudes. In addition, for both forms of intuitiveness, we find a significant and positive interaction effect with subjects’ prosocial attitudes on prosocial behavior. Hence, this study confirms previous findings from laboratory as well as online studies and provides external validity by demonstrating that the SHH applies in a real-life situation.

## Introduction

In many everyday situations as well as in many experimental studies, it has been observed time and again that a substantial proportion of people are willing to act prosocially, i.e., to forgo short-term benefits or incur some personal costs in order to improve the well-being of others [1–3]. These findings pose explanatory challenges in situations where mechanisms such as reputation or reciprocity cannot contribute to the emergence of prosocial behavior, i.e., interactions that are anonymous and one-time.

In the context of explaining prosocial behavior in one-time interactions, dual process theory (DPT) has proven to be particularly fruitful in recent years. According to the DPT, human cognition and action can only be explained by the interplay of two qualitatively distinct kinds of systems or types of mental processes, i.e., automatic-spontaneous (intuitive) processes are contrasted with controlled-deliberative (reflective) processes [4–6]. Automatic-spontaneous processes are typically automatic, fast, and associative, operate outside of the actor’s consciousness and in parallel. In contrast, controlled-deliberative processes are typically controlled, slow, and rule-based, operate within the actor’s consciousness and only in serial succession. While the exact interplay between these two kinds of processes is still a matter of debate [6, 7], DPT has nevertheless given rise to influential applications in research on prosocial behavior.

In particular, the idea that acting prosocially is an intuitive human behavior has received much attention. In short, this idea states that the more someone relies on his or her intuitiveness, i.e., the tendency to engage in an intuitive decision-making process, the more prosocially he or she will act [8, 9]. This hypothesis, which we will call the *intuitive prosociality hypothesis*, has been confirmed in many studies: First, in observational studies, where response latencies [8, 10, 11] and dispositional measures of thinking dispositions and cognitive styles [12] are used to assess subjects’ intuitiveness. Second, in experimental studies which aim at directly influencing subjects’ intuitiveness through procedures such as time pressure [8, 13, 14], cognitive load [15, 16], or ego-depletion [17].

At the same time, a number of skeptical contributions have appeared that question the empirical validity of the intuitive prosociality hypothesis on both theoretical as well as methodological grounds. In terms of methodology, it was argued that observational response latencies do not allow a straightforward inference about the type of cognitive process involved, because response latencies are also influenced, among other things, by the strength of preferences (discriminability of alternatives) [18, 19]. Some studies uncovered that intuition can have a positive influence on certain aspects of prosociality (e.g., egalitarian choices), while also having a negative influence on other aspects of prosociality (e.g., social efficient choices) [20, 21]. Further, it was also shown that by appropriately manipulating the payoff structure it can be observed that actors who tend to decide quickly behave less prosocially than actors who tend to decide slowly [18]. Moreover, even studies which employ experimental manipulations of the time available in decision-making do not necessarily yield results that support the intuitive prosociality hypothesis [22–24].

In light of this contradictory evidence, it became clear that the original hypothesis could not be upheld without additional qualifying statements. Thus, the *social heuristic hypothesis* (SHH) took its place [13]. The SHH consists of two parts: First, it states that humans internalize strategies that tend to be beneficial in their daily social life. These internalized strategies function as cues about how to behave in a new and unfamiliar social interaction. Second—and this is where DPT comes into play—the hypothesis states that actors who decide intuitively will follow these cues more closely than actors who reflect on the situation, thereby potentially recognizing that an alternative behavior is more advantageous.

The simplified idea behind this hypothesis is that decisions are less costly if they are made via an intuitive process rather than a reflective process. If the average cost saved by an intuitive decision (compared to the reflective decision) exceeds the average harm caused by this decision (in comparison to the reflective decision), intuitiveness may be evolutionarily stable [25–27]. Since prosocial behavior is—by definition—never advantageous in a one-time anonymous interaction, deliberation will undermine prosociality, whereas intuition might favor both cooperative as well as selfish behavior depending on prior experience. In other words, the nature of everyday social interactions is a moderating factor that influences the intuitive responses of actors. Assuming that it is true for most societies that prosocial behavior is more successful in daily life, the SHH can explain why intuitive actors might act more prosocially than their less intuitive counterparts. Furthermore, it can also explain why this might be not true for certain situations, societies, or parts thereof [12, 28].

In this paper, we focus on another potential moderator of the relationship between intuitiveness and prosocial behavior, namely prosocial attitudes [11, 29–31]. The basic idea is that prosocial attitudes, similar to previous experiences, also function as normative cues, but at the individual level. Therefore, the SHH can also be applied with respect to prosocial attitudes. Indeed, studies on this topic find that individuals with stronger prosocial attitudes act more prosocial. More interestingly, they also find evidence that prosocial individuals act less prosocial the longer they need to decide [29, 30, 32] and that similar results are observed when an experimental manipulation like time pressure is used [33].

The objective of this paper is to apply the SHH to a novel setting and investigate whether its implications hold true in a real-life situation. In doing so, we use a non-reactive field experiment in which subjects are unaware that they are part of a study. For this purpose, a *non-reactive field experiment* was conducted with subjects previously enrolled in a lab experiment at an Experimental Laboratory (LAB) [34]. The participants received an apparently misdirected email from the official email address of the lab, which contained a payoff code that allowed participants to redeem a monetary payment online. Since the code and the associated money were obviously intended for another person, we classify the attempt to redeem the code as less prosocial that simply ignoring the email. In addition, we classify both of these actions as less prosocial than the action of informing us of the alleged error. In this regard, we closely follow the psychological definition of prosociality, which refers to observed behavior, but not to underlying motivations [20, 35]. Thus, it is possible that an action we classify as prosocial might be driven by selfish rather than altruistic motives. Within the study, we experimentally varied three different parameters: First, the amount of money promised in the email, second, the verbal framing of the email, and third, the presence of a disclaimer. However, these experimental manipulations are only of secondary interest, as our main focus is on the interaction effects between attitudes and intuitiveness. For this purpose, we measured participants’ intuitiveness in two different ways: First, we asked participants in a follow-up survey to recall the situation in which they decided how to respond to the email and to self-assess their intuitiveness at this moment. A second measure indicating the participants’ general tendency toward intuitive behavior was collected in the previously conducted lab experiment using the so-called Cognitive Reflection Test [36]. Note that neither measurement is based on response time, which, as mentioned earlier, is often considered a problematic measure [18, 19]. In the lab experiment, we also obtained the prosocial attitude of the participants.

Following previous findings in the literature on attitudes and prosocial behavior [29, 30, 32], we expect that people with stronger prosocial attitudes will behave more prosocially. Furthermore, and in accordance with the SHH, we expect a positive interaction effect between prosocial attitudes and intuitiveness on prosocial behavior. Or, phrased in the opposite way, the negative effect of reflection (non-intuitiveness) on prosocial behavior should be most pronounced for subjects with strong prosocial attitudes and become less pronounced the weaker these attitudes are.

The remainder of the paper is organized as follows. In the next section the methods and the design of the field experiment are presented. The following section reports our empirical findings, and in the last section we draw conclusions with respect to the SHH and the DPT, discuss limitations of the current study, and outline directions for future research.

## Methods

In the style of the non-reactive lost letter method [37, 38], we developed a field experiment in which we sent apparently misdirected emails to which recipients could respond in different ways. The emails were sent from the official address of the Leipzig Experimental Laboratory (LAB) to former participants of a previous lab experiment conducted about six months before this study [34]. The field experiment was conducted in accordance with the Declaration of Helsinki and all procedures were approved by the Institute of Sociology of the Leipzig University.

The content of the emails was standardized and the alleged recipient of the email was a woman named “Marion Koch”. This name was chosen because, first, it is a relatively typical German name and, second, none of the recipients had either this first or last name, so it should be apparent to participants that they were not the intended recipients of the email. In the email, we thanked the recipients for allegedly participating in a lab experiment the day before, told them how much money they had earned in total, and sent them a payout code to receive the money anonymously (for the complete content of the email, see S1 Table). The recipients were told that the payout code had to be entered and submitted on the LAB website to be redeemed for an Amazon voucher, a PayPal credit, or a payout voucher by which the amount in cash could be anonymously received at the LAB. However, this website was prepared for the experiment in such a way that an error message appeared when the code was entered (“Unfortunately, this code has already been used.”) and the code, as well as the time of the attempt, were recorded.

For the experimental manipulation, we used a 2 × 2 × 2 between-participants factorial design. We varied the temptation, i.e., the amount of money Ms. Koch was supposedly entitled to, by indicating a payout amount of 6.25€ (temptation low treatment) and 21.75€ (temptation high treatment), respectively. Furthermore, the email either contained a disclaimer at the end (disclaimer treatment) or not (no-disclaimer treatment). The disclaimer informed the participants that this email was “intended exclusively for the person addressed” and that the LAB should be contacted if this email was not delivered to the rightful recipient. Finally, we varied the description of Ms. Koch’s action that had led to the earning of the money so that either it was described as “contribute to a group fund” (contribution treatment) or “steal from a group fund” (theft treatment).

The participants could respond in three possible ways: They could simply ignore the email, notify us of our “mistake”, or attempt to use the code. For simplicity, these behaviors will be referred to as neutral, helpful, and selfish actions in the following. In fact, there were some people who showed several responses, such as reporting the error first and using the code later. In such cases, we classified the person according to which response occurred first. This was possible because we had collected the date and time for each response.

At the end of the two-week data collection period, all participants were informed about the field experiment and invited to take part in an online follow-up survey. At this point, participants had the opportunity, in accordance with the Declaration of Helsinki, to object to their participation in the study. The primary goal of this follow-up survey was to measure the intuitiveness participants exhibited when they decided on how to respond to the email. This was done using a series of questions. For example, they were asked whether they had considered other alternative actions, how quickly they had made their decision, and to what extent they based their action on gut feeling (see S2 Table).

Since only persons who had previously participated in the lab experiment took part in the field experiment, we also have further information from the lab experiment at our disposal. In particular, two variables from the lab experiment are central to this study: First, the prosocial attitude of the participants, which is measured using a short version of the Prosocial Personality Battery [39] (see S3 Table), and second, the general intuitive tendency of the participants, which is measured using an extended version of the Cognitive Reflection Test (CRT) [36, 40]. The extended version of the CRT consists of the three original questions and one additional question [41] (see S4 Table). Following [12], we use the number of wrong answers in the CRT as a measure of a general intuitiveness tendency of the participants [20, 42]. In addition, age, gender, or lab experience serve as control variables.

## Results

A total of 763 participants of a previously conducted lab experiment could be reached by email and these constitute the participants of our field experiment. Of these, 19 individuals expressed suspicion during the data collection period that the email could be part of a study and were therefore excluded from further analyses. In addition, one of these individuals later decided to drop out of the study during the follow-up interview. Thus, a total of 744 valid observations are available for analysis. Of these, 35% and 65% report being male and female, respectively. The majority of our subjects (over 80%) are students and the average age is 25 years.

**Fig 1:**
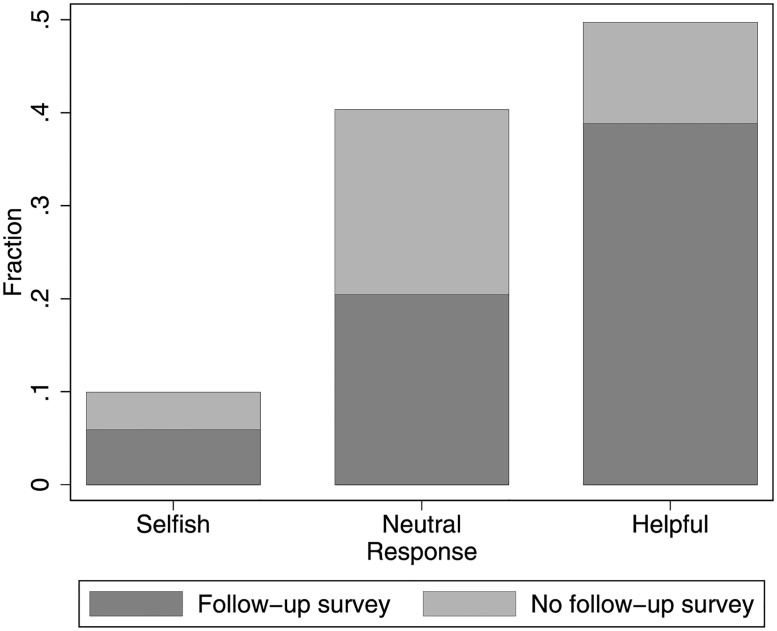
Overview response and participation. Distribution of participants’ responses in the field experiment and proportion of those who participated in the follow-up survey.

With respect to our dependent variable, i.e., the behavioral response to our email, we find that about half of the participants behaved helpfully and notify us of our alleged error, approximately 40% showed no reaction at all and are therefore classified as neutral, and about 10% of the participants behaved selfishly and attempt to enter the payout code online. In total, 485 subjects (65%) participated in the follow-up survey, with participation rates above 50% in each of the three response groups (Fig 1); however, three subjects did not answer all questions regarding the intuitiveness of their response, so only 482 complete cases are available.

**Fig 2:**
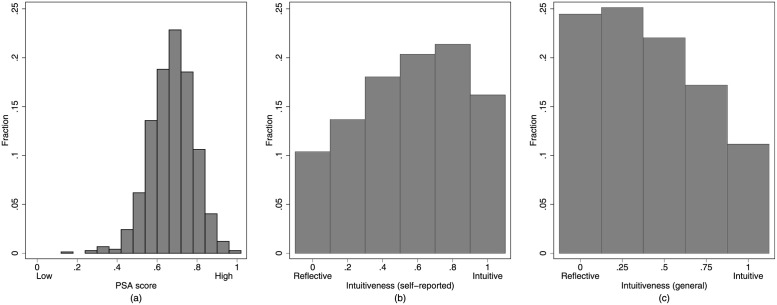
Overview main independet variables. Distribution of participants’ (a) PSA score, (b) self-reported intuitiveness, and (c) general intuitiveness.

Let us now turn to the central independent variables of the field experiment. As a measure of prosociality, we use an index based on the short version of the Prosocial Personality Battery. Therefore, we selected 13 questions of the Prosocial Personality Battery which maximize reliability (Cronbach’s *α* = .80) while minimizing the number of factors in a factor analysis (the selected questions can be found in S3 Table). This index can take values from 0 (= low prosocial attitudes) to 1 (= high prosocial attitudes) and will be referred to as the prosocial attitude (PSA) score. Fig 2(a) shows the distribution of the PSA score: on average, participants have a value of .677 with a standard deviation of .108. Less than 5% of the participants have a PSA score lower than 0.5, which means that we are dealing with a relatively prosocial sample in which there are hardly any participants with low prosocial attitudes. Therefore, if we were to compare two groups for the sake of illustration, it would be those with intermediate and high prosocial attitudes.

Regarding self-reported intuitiveness, we use an index calculated from the questions of the followup-survey. The index can take values from 0 (reflective) to 1 (intuitive) and has acceptable reliability with a Cronbach’s *α* of .70. The distribution of self-reported intuitiveness is a slightly left-skewed normal distribution (see Fig 2(b)). Furthermore, in Fig 2(c) we see a right-skewed distribution of the general tendency toward intuitive behavior (in the following: general intuitiveness), which is calculated using the number of wrong answers in the extended CRT. This measure can take values between 0 (reflective) and 1 (intuitive) and has a Cronbach’s *α* of .64.

Regarding possible correlations between our main independent variables, we find that the correlation between PSA score and general intuitiveness is not significant (*r* = .027, *p* = .460), while we observe a significant positive correlation between PSA score and self-reported intuitiveness (*r* = .106, *p* = .020). Hence, participants with higher prosocial attitudes are slightly more intuitive. Interestingly, the correlation between general intuitiveness and self-reported intuitiveness is negligibly weak (*r* = .039, *p* = .397).

In our analyses, we use participants’ first response in our field experiment as the dependent variable. The observed reactions can be ranked with respect to prosociality: Redeeming the code is the least prosocial action, followed by the neutral action and then by the helpful action of reporting the error. Thus, we use ordered-logit models (OLMs) to test our hypotheses. In these models, a positive regression coefficient of a variable indicates that an increase in that variable increases the probability of a more prosocial action, i.e., instead of the selfish action, the neutral or helpful action is chosen, or instead of the neutral action, the helpful action is chosen. Similarly, a negative coefficient indicates that an increase in this variable decreases the likelihood of a prosocial action. However, an important prerequisite for using ordered logit models is the proportional odds assumption, which states that the calculated odds ratios must be the same for each of the ordered dichotomizations of the dependent variable [43]. To check whether this assumption is warranted, we used the Brant test [44]. It turned out that none of our models violates this assumption. As a test of robustness, we performed analogous analyses using OLS models. All variables which are significant under the OLM specification prove to be significant under the OLS specification as well.

**Fig 3:**
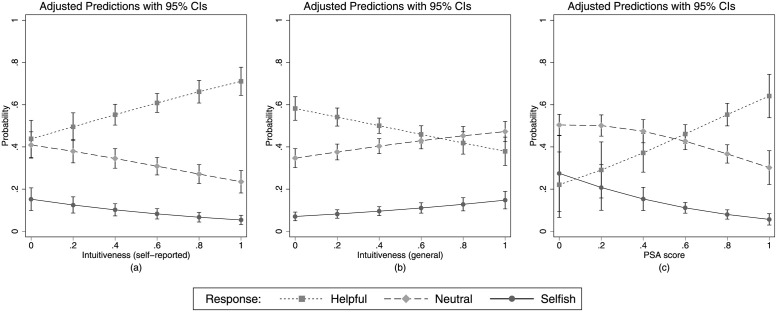
Bivariate analyses. Predicted probabilities of behavioral responses as a function of (a) self-reported intuitiveness, (b) general intuitiveness, and (c) PSA score.

Consistent with the intuitive prosociality hypothesis, in the bivariate analysis (see Fig 3(a)) we find that a significant positive effect on prosociality can be observed for self-reported intuitiveness (*β* = 1.148, *p* < .001). However, the opposite is true for the general tendency towards intuitive behavior (Fig 3(b)); we observe that individuals tend to be less prosocial the higher their general intuitiveness (*β* = −.824, *p* < .001). Finally, as previous studies have shown [29, 33], we observe a significant and positive effect of the prosocial attitude on prosocial behavior (*β* = 1.841, *p* = .006, Fig 3(c)).

To test the interaction effects between intuitiveness and the PSA score as posited by the SHH, we estimate two OLMs, which can be found in Table 1. Consistent with the SHH, we observe a significant positive interaction effect of the PSA score with self-reported intuitiveness in Model 1 (*β* = 6.049, *p* = .021). That is, an increase in (self-reported) intuitiveness has a greater impact on the probability of prosocial behavior for participants with stronger prosocial attitudes than for participants with less pronounced prosocial attitudes. In addition, we find that intuitiveness does not promote prosocial behavior for participants with low prosocial attitudes (*β* = −2.943, *p* = .095). Furthermore, we find that among perfectly reflective participants variations in prosocial attitudes do not affect prosocial behavior significantly (*β* = −1.383, *p* = .399). Similarly, Model 2 shows the interaction between the PSA score and general intuitiveness; there is a highly significant and positive interaction effect (*β* = 5.740, *p* = .005). Again, we find that general intuitiveness does decrease prosocial behavior for participants without a strong attitude towards prosociality (*β* = −4.709, *p* < .001). It is also observed that among participants with an extreme tendency towards cognitive reflection variations in prosocial attitudes have no impact on prosocial behavior (*β* = −.329, *p* = .753).

**Table 1:** Ordered logit regression models of behavioral responses in the field experiment.

*Response*	Model 1		Model 2		Model 3	
	Coef.	SE	Coef.	SE	Coef.	SE
PSA score	−1.383	1.639	−.329	1.045	−3.388^+^	1.956
Intuitiveness (self-rep.)	−2.943^+^	1.761			−2.541	1.858
PSA × Int. (self-rep.)	6.049*	2.631			5.626*	2.749
Intuitiveness (general)			−4.709***	1.391	−4.983**	1.857
3lPSA × Int. (general)			5.740**	2.037	6.171*	2.741
Disclaimer treatment					.350^+^	.191
Theft treatment					.165	.189
High temptation					.152	.189
Male gender					−.155	.210
Age					.342^+^	.196
Age^2^					−.006^+^	.004
Naive					.029	.204
McFadden’s pseudo *R*^2^	.030		.022		.061	
*N*	482		744		482	

^+^ *p* < .1,

* *p* < .05,

** *p* < .01,

*** *p* < .001.

**Fig 4:**
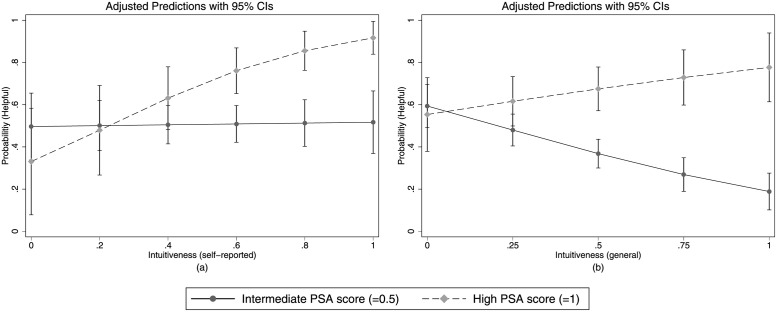
Multivariate analyses. Predicted probabilities of the prosocial action as a function of PSA score and either (a) self-reported intuitiveness or (b) general intuitiveness.

Panel (a) and (b) of Fig 4 show the predicted probabilities of behaving prosocially according to Model 1 and Model 2, respectively. For convenience, only the probabilities of the prosocial action and only individuals with an intermediate (= 0.5) or high (= 1) PSA score are shown. Both models predict that participants with high prosocial attitudes behave more prosocially the more intuitive they are, whereas participants with an intermediate PSA score are either not affected by their intuitiveness or behave even less prosocially the greater their tendency towards intuition.

**Fig 5:**
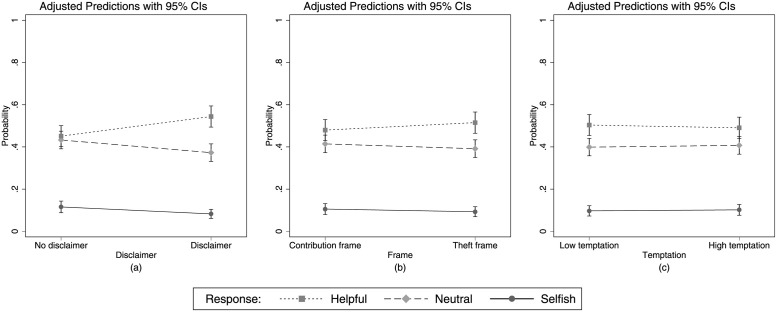
Experimental manipulations. Predicted probabilities of behavioral responses as a function of experimental manipulations.

Let us now turn to the experimental manipulations regarding the content of the email. As before, we use bivariate OLMs for the analyses. We observe that the disclaimer makes it significantly more likely that a more prosocial action is chosen (*β* = .372, *p* = .009, Fig 5(b)). We further observe, that neither the framing of how the imaginary person earned their money (*β* = .141, *p* = .317, Fig 5(b)) nor the amount of money (*β* = −.052, *p* = .712, Fig 5(c)) had any significant effect on the behavior of the participants.

Finally, we combine Model 1 and 2 to perform an additional test for robustness and to check whether both intuitiveness measures have independent effects (Table 1). For this purpose, Model 3 includes the variables of the previous models and additionally controls for our treatment variables (treatment not used = 0 / used = 1). Also, we control for gender (female = 0 / male = 1), previous lab experience (no = 0 / yes = 1), age in years, and squared age.

Our main results of the previous analyses can be replicated in this extended model. Most importantly, we find significant interaction effects between both measures of intuitiveness and the PSA score. Again, neither self-reported nor general intuitiveness promote prosocial behavior among participants with low prosocial attitudes. On the contrary, we even find a significant negative effect of general intuitiveness on prosocial behavior among subjects with an intermediate PSA score. Regarding the experimental manipulations of the content of the email, we observe that the disclaimer treatment has a weakly significant and positive effect on the prosocial behavior of our participants, whereas the theft treatment and the high temptation treatment exhibit no significant effects.

In addition, Model 3 shows that prosocial attitudes have a weakly significant negative effect on prosocial behavior among participants which are extremely reflective according to both measures of intuitiveness. However, this result should not be overestimated, because it is not robust under alternative model specifications.

With respect to the control variables, we observe no significant effect of gender or previous lab experience and only a weakly significant positive effect of age and a weakly significant negative effect of squared age.

### Robustness checks

At this point, we perform various robustness checks, to assess whether our results are also valid under other assumptions. First, since the adequacy of the CRT as a measure of intuitiveness has been criticized [45], we also use the number of intuitively answered questions of the extended CRT as an alternative measure for general intuitiveness [12]. We recalculate all models with this alternative index of general intuitiveness and find that all main effects remain virtually unchanged except for the PSA score in the full model which turns insignificant (S5 Table).

Second, since our research interest is focused on interaction effects and these could potentially be biased in the context of logit models [46], we follow standard practice and additionally test our models using linear regression [47]. We observe that the linear models are in line with our previous findings (S6 Table).

Finally, we deal with the problem that is central to many non-reactive studies: How can we ensure that subjects did not suspect they might be part of a study? As mentioned at the beginning of this section, we excluded all subjects from the study who had already expressed suspicion during the field phase. In addition, we asked the participants of the follow-up survey whether they had any suspicions before or during their reaction to the email. A total of 82 subjects indicated that they had developed suspicions before or during their reaction. However, because these subjects do not behave significantly different from those not expressing suspicion in the follow-up survey (*χ*^2^ = 3.702, *p* = 0.157), we did not remove them from the main analysis. But even if we remove these subjects from the study and recalculate our models, we find no major changes (S7 Table). Altogether, we can say that our results are robust to a number of alternative models.

## Discussion

In this paper, we report results of a non-reactive field experiment to contribute to the discussion regarding the social heuristic hypothesis (SHH) [13]. A notable feature of the current study is that the participants of the field experiment had previously participated in a lab experiment which is why we have solid measures of participants’ prosocial attitudes as well as their general intuitiveness at our disposal. In addition, we work with two measures of intuitiveness, one based on self-reports in a follow-up survey and the other one based on an extension of the Cognitive Reflection Test [36].

In line with our expectations and previous studies [29, 30, 32], we observe that participants with higher prosocial attitudes behave more prosocial. While we find that self-reported intuitiveness has a positive effect on prosocial behavior in a bivariate analysis, this effect vanishes as soon as we control for prosocial attitudes. In accordance with other studies [12], we find that general intuitiveness has a negative effect on prosocial behavior even in bivariate analysis. What we do find with respect to both self-reported as well as general intuitiveness in multivariate analyses is that prosocial attitudes have a greater impact on prosocial behavior among participants with a greater tendency towards intuitiveness, which is in line with the literature on the SHH and prosocial attitudes [29, 30]. This finding is robust regardless of whether we look at each measure of intuitiveness separately or simultaneously and regardless of whether we work with control variables or not.

All in all, our study contributes to the growing literature that puts serious doubts on the empirical validity of the simplistic hypothesis of intuitive prosociality. As we saw, individual heterogeneity (in our case with respect to internalized attitudes) might be an important factor to consider when studying the interplay of intuitiveness and prosociality. In particular, our results confirm the more nuanced and complex implication of the SSH according to which the influence of intuitiveness on prosociality is moderated by strength and type of internalized attitudes. Furthermore, we were able to show that the SHH also applies beyond the controlled environment of laboratory studies to a more realistic situation of everyday life.

Regarding the experimental manipulations, on the one hand, we observe that a subtle cue like a disclaimer can influence subjects’ responses. On the other hand, the description of how Ms. Koch got her money seems too subtle to cause a behavioral change. In addition, we observe that the incentive associated with the use of the code does not affect prosocial behavior. Taken on face value, this finding contradicts the idea that behavior should be responsive to incentives. However, the finding can also be interpreted differently against the background of experimental literature on lying and cheating [48, 49]. Regarding the act of deception, [50] shows that while increasing the gain for the actor increases the probability of deception, increasing the harm to another person decreases this probability. Since in our field experiment both potential gain and harm were de facto manipulated simultaneously, the effects of these two kinds of incentives could neutralize each other, which might explain why we observe no effect.

Turning to the limitations of the current study, we first discuss a peculiar drawback of our non-reactive design. With respect to participants who showed a neutral reaction, we have no way of knowing whether they actually got the email or not. As a consequence, the fraction of neutral reactions to our email is potentially biased upwards. While we acknowledge this drawback, we do not believe that it seriously diminishes our qualitative findings, for several reasons. First, note that only our analyses working with the general measure of intuitiveness are affected by this potential problem. The results with respect to the self-reported measure only refer to subjects who participated in the follow-up survey and were consequently able to get the email from our lab. Also, unreported, additional analyses show that our results regarding general intuitiveness are robust if we restrict the analyses to those subjects who participated in the follow-up survey. Second, there are good reasons to believe that the fraction of participants who did not react to the email because they have not read it, is actually rather small. For one, almost all of our participants were students who subscribed to the email list of the lab voluntarily, most likely to earn some extra cash. Against this background, it is reasonable to expect our participants to be rather attentive towards emails from the lab. Also, the participation rates regarding the follow-up survey do not differ too much between participants who showed no reaction and those who showed a selfish or helpful reaction.

Other limitations of the current study are related to the special composition of the sample and the measurements of intuitiveness. First, there are almost no participants with low prosocial attitudes. As a consequence, we could not test the interesting hypothesis that low prosocial attitudes have a greater impact on selfish behavior among participants with a stronger tendency towards intuitiveness. Future research should work with a more diverse sample to remedy this deficit. Finally, it was a bit surprising to discover that our two measures of intuitiveness do not correlate at all. While it can even be interpreted as an advantage, because our results are robust with respect to two independent measures of intuitiveness, it raises the question to what extent the measure based on self-reports is actually reliable. In any case, it is a worthwhile task for future research to check whether our findings regarding the interaction between prosocial attitudes and intuitiveness are robust if intuitiveness is not measured but experimentally manipulated, for instance via time pressure or cognitive overload.

## Supporting information

S1 Data (CSV)

S1 Table Email text. The text of the email translated from German. Text passages in square brackets mark experimental manipulations.
(PDF)

S2 Table Self-reported intuitiveness. German translations and scales of questions to determine the intuitiveness of the decision in the field experiment.
(PDF)

S3 Table Short Prosocial Personality Battery. All responses to these questions were answered on a 5-point Likert scale ranging from 1 “Strongly Disagree” to 5 “Strongly Agree“.
(PDF)

S4 Table Extended cognitive reflection test. (PDF)

S5 Table Alternative ordered logit regression models. Using the alternative measure for general intuitiveness.
(PDF)

S6 Table Linear regression models. The response is treated as quasi metric ranging from -1 (selfish) to 0 (neutral) to 1 (helpful).
(PDF)

S7 Table Ordered logit regression models. Without subjects that stated suspicion in the follow-up survey.
(PDF)
