# Supplementary material for: Intuition, reflection, and prosociality: Evidence from a field experiment
Source: PLoS One. 2022 Feb 25;17(2):e0262476. doi: 10.1371/journal.pone.0262476 (PMC8880868; doi:10.1371/journal.pone.0262476)
Supplement: S1 Table — The text of the email translated from German. Text passages in square brackets mark experimental manipulations. (PDF) [file pone.0262476.s002.pdf]

Dear Ms. Marion Koch,<br>thank you again for your participation yesterday (February 7, 2019) in our LAB lab study. The study examines conditions under which actors [contribute to][steal from] a group fund, thereby making their group [better off] [worse off] as a whole. We have since received the decisions of the other participants and were able to determine your payout amount. Taking into account the participation bonus and the amount you have [contributed to][stolen from] the group fund, your total payout is [EUR 6.25][EUR 21.75].<br>To receive the money is quite simple. You first need your personal payout code, under which we have anonymously stored the above payout. You will receive this code with this email. Your personal payout code is: <CODE><br>Follow our link to the LAB website <LINK> and enter your payout code. There you can choose whether you want to collect the money anonymously via pickup slip at the LAB, receive it as Amazon voucher or via PayPal transfer. If you have any questions about this procedure, we are always available by email.<br>Please note that you must redeem your payout code no later than February 22, 2019! Unfortunately, a later encashment of the payout code is not possible.<br>Best regards,<br>Your LAB Team<br>[][This email contains information intended exclusively for the person addressed. This also applies to any attached documents. The information may be confidential and/or exempt from publication. Copying and disclosure to unauthorized third parties is prohibited. If you are not the recipient, please notify the sender and delete this email from your system.]

S1 Table. Email text. The text of the email translated from German. Text passages in square brackets mark experimental manipulations.
