# Supplementary material for: Intuition, reflection, and prosociality: Evidence from a field experiment
Source: PLoS One. 2022 Feb 25;17(2):e0262476. doi: 10.1371/journal.pone.0262476 (PMC8880868; doi:10.1371/journal.pone.0262476)
Supplement: S2 Table — German translations and scales of questions to determine the intuitiveness of the decision in the field experiment. (PDF) [file pone.0262476.s003.pdf]

Question	Scale	Index
Did you think, at least in brief, about behaving differently than you ultimately did?	yes / no	yes $\Rightarrow$ 0, no $\Rightarrow$ .2
How much time elapsed between reading the email and your decision on how to deal with it?	minutes	Median Split: ≥ 4 minutes $\Rightarrow$ 0, < 4 minutes $\Rightarrow$ .2
I had a hard time deciding how to respond to the email.	5-item Likert-scale: 1 = Does not apply at all 5 = Fully applies	Median Split: $\geq 3 \Rightarrow 0$, $< 3 \Rightarrow$ .2
I made a spontaneous gut decision.		Median Split: $\geq 5 \Rightarrow .2$, $< 5 \Rightarrow$ 0
I carefully weighed the various possible courses of action.		Median Split: $\geq 3 \Rightarrow 0$, $< 3 \Rightarrow$ .2

S2 Table. Self-reported intuitiveness. German translations and scales of questions to determine the intuitiveness of the decision in the field experiment.
