# Supplementary material for: Intuition, reflection, and prosociality: Evidence from a field experiment
Source: PLoS One. 2022 Feb 25;17(2):e0262476. doi: 10.1371/journal.pone.0262476 (PMC8880868; doi:10.1371/journal.pone.0262476)
Supplement: S3 Table — All responses to these questions were answered on a 5-point Likert scale ranging from 1 “Strongly Disagree” to 5 “Strongly Agree“. (PDF) [file pone.0262476.s004.pdf]

| Question |
| --- |
| When I see someone being taken advantage of, I feel kind of protective towards them. |
| I sometimes try to understand my friends better by imagining how things look from their perspective. |
| Other people's misfortunes do not usually disturb me a great deal. |
| When I see someone being treated unfairly, I sometimes don't feel very much pity for them. |
| I am often quite touched by things that I see happen. |
| I believe that there are two sides to every question and try to look at them both. |
| When I'm upset at someone, I usually try to "put myself in their shoes" for a while. |
| My decisions are usually based on my concern for other people. |
| My decisions are usually based on what is the most fair and just way to act. |
| I choose alternatives that are intended to meet everybody's needs. |
| I choose a course of action that maximizes the help other people receive. |
| I choose a course of action that considers the rights of all people involved. |
| My decisions are usually based on concern for the welfare of others. |

**S3 Table. Short Prosocial Personality Battery.** All responses to these questions were answered on a 5-point Likert scale ranging from 1 "Strongly Disagree" to 5 "Strongly Agree".
