# Supplementary material for: Intuition, reflection, and prosociality: Evidence from a field experiment
Source: PLoS One. 2022 Feb 25;17(2):e0262476. doi: 10.1371/journal.pone.0262476 (PMC8880868; doi:10.1371/journal.pone.0262476)
Supplement: S4 Table — (PDF) [file pone.0262476.s005.pdf]

Question	Correct answer	Intuitive answer
A bat and a ball cost 1.10€ in total. The bat costs 1.00€ more than the ball. How much does the ball cost?	.05	.10
If it takes 5 machines 5 minutes to make 5 widgets, how long would it take 100 machines to make 100 widgets?	5	100
In a lake, there is a patch of lily pads. Every day, the patch doubles in size. If it takes 48 days for the patch to cover the entire lake, how long would it take for the patch to cover half of the lake?	47	24
If you are running in a race and pass the person in second place, what position are you in?	2	1

S4 Table. Extended Cognitive Reflection Test.
