# Supplementary material for: Intuition, reflection, and prosociality: Evidence from a field experiment
Source: PLoS One. 2022 Feb 25;17(2):e0262476. doi: 10.1371/journal.pone.0262476 (PMC8880868; doi:10.1371/journal.pone.0262476)
Supplement: S5 Table — Using the alternative measure for general intuitiveness. (PDF) [file pone.0262476.s006.pdf]

	Model 2b		Model 3b	
Response	Coef.	SE	Coef.	SE
PSA score	.187	.932	−3.081	1.906
Intuitiveness (self-rep.)			−2.420	1.882
PSA × Int. (self-rep.)			5.477*	2.780
Intuitiveness (intuitive CRT answers)	−4.794**	1.466	−5.834***	1.988
PSA × Int. (intuitive CRT answers)	5.656**	2.146	7.185*	2.927
Disclaimer treatment			.323+	.191
Theft treatment			.152	.189
High temptation			.142	.190
Male gender			-.150	.209
Age			.355+	.195
Age^2			-.007+	.004
Naive			.018	.204
McFadden's pseudo R^2	.023		.064	
N	744		482	

$^+p < .1$, $^*p < .05$, $^{**}p < .01$, $^{***}p < .001$.

S5 Table. Alternative ordered logit regression models Using the alternative measure for general intuitiveness.
