# Supplementary material for: Intuition, reflection, and prosociality: Evidence from a field experiment
Source: PLoS One. 2022 Feb 25;17(2):e0262476. doi: 10.1371/journal.pone.0262476 (PMC8880868; doi:10.1371/journal.pone.0262476)
Supplement: S6 Table — The response is treated as quasi metric ranging from -1 (selfish) to 0 (neutral) to 1 (helpful). (PDF) [file pone.0262476.s007.pdf]

	Model 1c		Model 2c		Model 3c	
Response	Coef.	SE	Coef.	SE	Coef.	SE
PSA score	$-.376$	.524	$-.035$	.331	-1.018^{+}	.575
Intuitiveness (self-rep.)	$-.821$	.555			$-.785$	.553
PSA × Int. (self-rep.)	1.740^{*}	.812			1.708^{*}	.808
Intuitiveness (general)			-1.574^{***}	.456	-1.635^{***}	.558
PSA × Int. (general)			1.926^{**}	.669	2.042^{*}	.815
Disclaimer treatment					.083	.059
Theft treatment					.038	.058
High temptation					.043	.058
Male gender					$-.032$	.064
Age					$.099^{*}$	.042
Age^2					$-.002^{*}$	.001
Naive					$-.001$	.062
McFadden's R^2	.051		.041		.102	
N	482		744		482	

$^{+}p < .1$,$^{*}p < .05$, $^{**}p < .01$, $^{***}p < .001$.

S6 Table. Linear regression models The response is treated as quasi metric ranging from -1 (selfish) to 0 (neutral) to 1 (helpful).
