# Supplementary material for: Intuition, reflection, and prosociality: Evidence from a field experiment
Source: PLoS One. 2022 Feb 25;17(2):e0262476. doi: 10.1371/journal.pone.0262476 (PMC8880868; doi:10.1371/journal.pone.0262476)
Supplement: S7 Table — Without subjects that stated suspicion in the follow-up survey. (PDF) [file pone.0262476.s008.pdf]

	Model 1d		Model 2d		Model 3d	
Response	Coef.	SE	Coef.	SE	Coef.	SE
PSA score	−1.535	1.939	−.354	1.138	-3.893^{+}	2.291
Intuitiveness (self-rep.)	−3.181	2.022			−2.703	2.080
PSA × Int. (self-rep.)	6.457^{*}	3.001			5.901^{+}	3.079
Intuitiveness (general)			-4.616^{**}	1.477	-5.098^{*}	2.028
PSA × Int. (general)			5.708^{**}	2.160	6.472^{*}	2.993
Disclaimer treatment					$.365^{+}$	.211
Theft treatment					.311	.211
High temptation					.218	.209
Male gender					−.142	.237
Age					.381	.297
Age^2					−.008	.005
Naive					−.083	.229
McFadden's pseudo R^2	.033		.021		.064	
N	400		662		400	

$^{+}p < .1$, $^{*}p < .05$, $^{**}p < .01$, $^{***}p < .001$.

S7 Table. Ordered logit regression models. Without subjects that stated suspicion in the follow-up survey.
